# Spectral Tuning Mechanism of Primate Blue-sensitive Visual Pigment Elucidated by FTIR Spectroscopy

**DOI:** 10.1038/s41598-017-05177-4

**Published:** 2017-07-07

**Authors:** Kota Katayama, Yuki Nonaka, Kei Tsutsui, Hiroo Imai, Hideki Kandori

**Affiliations:** 1 Department of Life Science and Applied Chemistry, Nagoya Institute of Technology, Showa-ku, Nagoya 466-8555 Japan; 2 OptoBioTechnology Research Center, Nagoya Institute of Technology, Showa-ku, Nagoya 466-855 Japan; 3 Primate Research Institute, Kyoto University, Inuyama, 484-8506 Japan

## Abstract

Protein-bound water molecules are essential for the structure and function of many membrane proteins, including G-protein-coupled receptors (GPCRs). Our prior work focused on studying the primate green- (MG) and red- (MR) sensitive visual pigments using low-temperature Fourier transform infrared (FTIR) spectroscopy, which revealed protein-bound waters in both visual pigments. Although the internal waters are located in the vicinity of both the retinal Schiff base and retinal β-ionone ring, only the latter showed differences between MG and MR, which suggests their role in color tuning. Here, we report FTIR spectra of primate blue-sensitive pigment (MB) in the entire mid-IR region, which reveal the presence of internal waters that possess unique water vibrational signals that are reminiscent of a water cluster. These vibrational signals of the waters are influenced by mutations at position Glu113 and Trp265 in Rh, which suggest that these waters are situated between these two residues. Because Tyr265 is the key residue for achieving the spectral blue-shift in λ_max_ of MB, we propose that these waters are responsible for the increase in polarity toward the retinal Schiff base, which leads to the localization of the positive charge in the Schiff base and consequently causes the blue-shift of λ_max_.

## Introduction

Vision constitutes 80% of human sensory information. One important function of vision is color discrimination. Many vertebrates share this sense and have evolved to adapt to different life environments, such as blue, green, and red (RGB) color perception in humans and more than four types of color perception in birds, fishes, insects, and crustaceans^1–3^. A remaining issue is how humans distinguish this wide range of wavelengths using the same chromophore. Four types of light-sensitive molecules and visual pigments exist in our retina: rhodopsin (Rh), which is responsible for the highly sensitive twilight vision, and three cone pigments that mediate color discrimination. Each cone pigment absorbs blue (λ_max_ = 425 nm), green (λ_max_ = 530 nm) or red (λ_max_ = 560 nm) light^4–6^, which corresponds to our RGB primary colors. All pigments possess the common chromophore 11-*cis*-retinal, which is covalently bound to the protein moiety. The specific interaction between 11-*cis*-retinal and the protein moiety determines the absorption of various wavelengths of light^7^. In spectral tuning, chromophore-protein interactions include (I) chromophore distortion, (II) electrostatic interactions between the protonated Schiff base and its specific counterion amino acid, e.g., glutamic acid at position 113 in Rh, and (III) polarity modulation around the β-ionone ring and polyene chain^7^.

For G protein-coupled receptors (GPCRs), the largest family of membrane proteins that mediate most of our cellular responses (including vision) are protein-bound internal water molecules, which play a critical role in protein stabilization, ligand recognition and G protein activation^8, 9^. Note that water molecules also play an active role in modulating the polarity of their ligand binding pocket^7^.

Fourier transform infrared (FTIR) spectroscopy has been extensively used to investigate not only the chromophore structure and chromophore-protein interactions but also protein-bound water molecules^10–13^. Previously, we reported light-induced differences in the FTIR spectra of primate green- (MG) and red- (MR) sensitive pigments at 77 K, which provided direct evidence of the presence of internal water molecules in the retinal binding pocket and new insight into the spectral tuning mechanism^14–16^. The observed vibrational signals of the water molecules in MG and MR were slightly different from each other, and these water molecules were proposed to be situated in the β-ionone ring region of the chromophore. Thus, protein-bound water molecules around the β-ionone ring may also be responsible for the 30 nm difference in λ_max_ between MG and MR.

Herein, we report the first difference in the FTIR spectra of the primate blue-sensitive pigment (MB) that contains vital information regarding the retinal chromophore, protein moiety, and internal water molecules. In comparison with other pigments, MB displayed significant differences in its retinal conformation and the peptide-backbone structure of α-helices. Additionally, the unique hydrogen-bonding network of water was found in the vicinity of 11-*cis*-retinal. In contrast, previous site-directed mutagenesis studies have reported that several mutations in the vicinity of the retinal chromophore of the human blue-sensitive pigment (HB) induced a spectral shift in λ_max_^17, 18^. Among them, Y265W in HB resulted in a significant red-shift of approximately 10 nm in λ_max_^18^. Then, in our report, mutation studies, which were focused on the spectral blue shift in λ_max_ of MB (like Y265W and/or E113D), revealed a dipole effect in the specific internal water cluster. Unique retinal-protein interactions can be clarified using the present comprehensive vibrational spectral analysis of cone pigments.

## Results and Discussion

### Batho minus MB, MRh, MG, and MR infrared spectra in the 1800–800 cm^−1^ region

**Figure 1:**
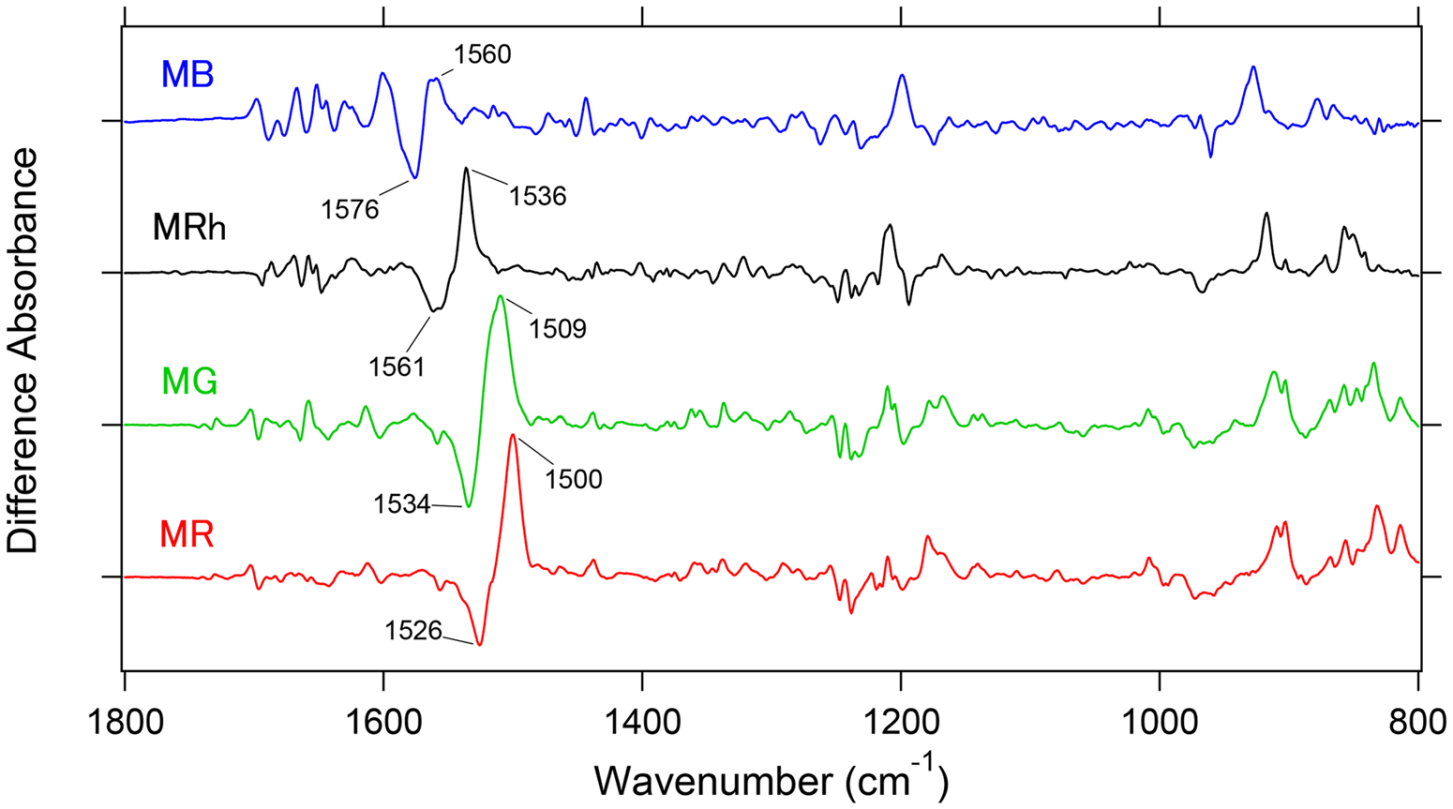
Light-induced difference FTIR spectra of MB, MRh, MG and MR in the 1800–800 cm^−1^ region at 77 K. Light-induced all-*trans* minus 11-*cis* difference FTIR spectra of MB, MRh, MG and MR in the 1800–800 cm^−1^ region measured at 77 K in D_2_O. The negative bands originate from the 11-*cis* form in the unphotolyzed state, whereas the positive bands originate from the all-*trans* form (batho-intermediate; Batho) after retinal photoisomerization. One division of the y-axis corresponds to 0.0015 absorbance units. A pair of bands at 1600–1500 cm^−1^ for each pigment corresponds to the ethylenic C=C stretching vibration of the retinal chromophore, and their spectral down-shifts correspond to the red-shift in the visible region.

Figure 1 shows a comparison of all-*trans* minus 11-*cis* difference FTIR spectra that correspond to primary structural changes in the visual excitation of each cone pigment. The overall spectral features of MB are similar to that of MRh, MG, and MR, although they share ~50% of the sequence identity in the 26 amino acid residues that surround the retinal chromophore (Figure S1)^5^. A pair of peaks at 1576 (−)/1560 (+) cm^−1^ corresponds to the ethylenic C=C stretching vibration of MB, and a spectral down-shift in this vibrational mode of the retinal chromophore at 1580–1500 cm^−1^ corresponds to a red-shift in the visible region^7^, which is evident from the linear correlation between λ_max_ and the negative frequency of the 11-*cis* form (Figure S2d).

**Figure 2:**
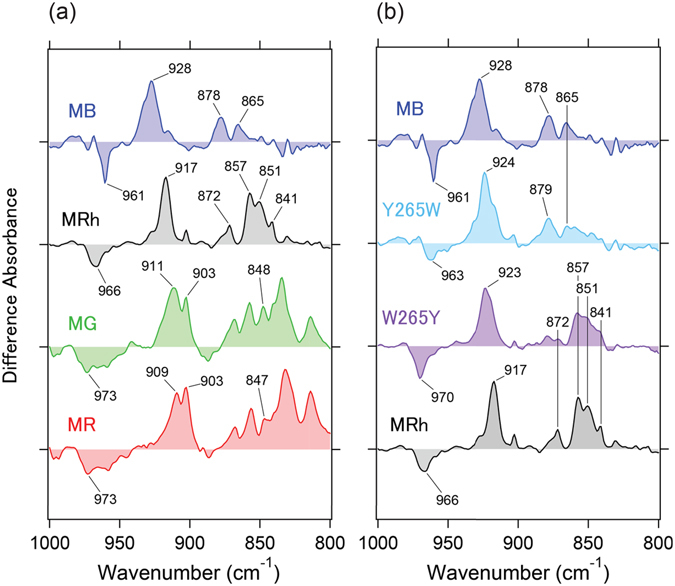
Spectral comparison of the mutant protein in the 1000–800 cm^−1^ region. (**a**) Expanded spectra from Fig. 1 in the 1000–800 cm^−1^ region. One division of the y-axis corresponds to 0.0009 absorbance units. (**b**) Light-induced all-*trans* minus 11-*cis* difference FTIR spectra of MB, MB-Y265W, MRh-W265Y and MRh in the 1000–800 cm^−1^ region measured at 77 K in D_2_O. One division of the y-axis corresponds to 0.0009 absorbance units.

The retinal chromophore exhibits hydrogen out-of-plane (HOOP) wagging modes in the frequency region of 1000–800 cm^−1^ and reflects a structurally perturbed and/or distorted chromophore. Previous resonance Raman spectroscopic studies for rhodopsin reported that C_11_=C_12_ HOOP modes of the dark state appeared at 969 cm^−1^ ^19^. In contrast to the vibrational modes of rhodopsin, the batho-intermediate state provided two bands: C_11_H wagging at 921 cm^−1^ and C_12_H wagging at 858 cm^−1^ ^19–21^. This is in part because the strongly coupled double bond at C_11_ and C_12_ of the retinal polyene chain are decoupled by photoisomerization, in which a specific geometric distortion occurred. This interpretation is consistent with the structural comparison between rhodopsin^22^ and the batho-intermediate state^23^ (Figure S3a). Figure 2a shows the HOOP mode region of the retinal chromophore. In MB, C_11_=C_12_ HOOP modes of the dark state appear at 961 cm^−1^ and C_11_H and C_12_H wagging modes of the corresponding batho-intermediate state emerge at 928 and 865 cm^−1^, respectively. Note that the frequency of the C_11_H wagging mode is linearly correlated with λ_max_ of each pigment, including the (Trp/Tyr) 265 mutants of both MB and MRh (Figs 2 and S4). Generally, HOOP modes are not correlated with absorption because they monitor local out-of-phase modes that intensify upon retinal geometric distortion as described above^19–21^. However, this observation indicates that a highly distorted C_11_ position influences the electronic state that leads to vibrational frequency changes. In fact, previous Raman spectroscopy studies suggest that the electrostatic interaction between the protonated Schiff base and counterion is relevant to the perturbation of C_11_=C_12_ HOOP vibrational modes, in which electrostatic interactions also play an important role in the spectral tuning as discussed later^24^.

Band shapes of coupled C_11_=C_12_ HOOP modes for each pigment are clearly different in the dark states, as shown in Fig. 2. Notably, the spectral features of MB are sharper relative to MG and MR (Fig. 2, left panel). Figure S4c presents the relationship between the band area of the C_11_=C_12_ HOOP mode and λ_max_ for each pigment, which shows that the shorter wavelength absorbing pigments have smaller band areas. This result reveals that the C_11_=C_12_ HOOP mode of MB represents the strongest coupling, namely, a double bond at the C_11_ and C_12_ positions that retains its geometric planar structure compared with MG and MR in their dark states. Alternatively, the extent of decoupling of the C_11_=C_12_ HOOP mode upon photoisomerization reflects the geometric distortion of the retinal chromophore and thus can be interpreted as frequency differences between the splitting C_11_H and C_12_H wagging modes. Figure S4d and Table 1 compares the positions of the HOOP bands for C_11_H and C_12_H in the batho-intermediate states and the frequency difference values between them. Remarkably, all WT and mutated pigments display a similar pattern, which indicates both a similar distortion in the bound all-*trans* retinal and a common *cis*-*trans* isomerization mechanism.

For Rh, the formation of the batho-intermediate state occurs on an ultrafast timescale of 200 fs with an extremely high quantum yield of 0.67^25, 26^. This unique photochemical event was significantly affected by 1) the structure of the retinal binding pocket, 2) the retinal structural planarity or 3) a combination of both factors. Previous femtosecond-stimulated Raman spectroscopy measurements revealed that geometric changes that were involved in the photoisomerization of the retinal chromophore occurred on the ground potential surface from the electronically excited state to batho-intermediate state of Rh, which enabled a fast reaction rate on an approximate 200 fs timescale^27^. Additionally, the main alteration was only observed in the retinal conformation from the twisted 11-*cis* form in the dark state to the extended all-*trans* form^22, 23^. However, only minor changes in the retinal binding pocket were observed from the crystal structures of the dark and batho-intermediate states of Rh^22, 23^. Therefore, the chromophore structure is the most important attribute for the ultrafast photoisomerization and high quantum yield of the isomerization of Rh. However, our results indicate that the retinal conformation of MB, especially the polyene chain, retains its planar structure, unlike MRh, MG, and MR. Therefore, the environment of the retinal binding pocket also contributes to efficient retinal photoisomerization of MB. In fact, previous photocurrent measurements in photoreceptors of larval salamanders (not primates) provided similar photosensitivities of Rh, and red- and blue-sensitive cone pigments, which strongly indicate that cone pigments also have high quantum efficiencies of photoisomerization such as Rh (although the actual values of those quantum yields of retinal photoisomerization have not been determined)^28^.

**Figure 3:**
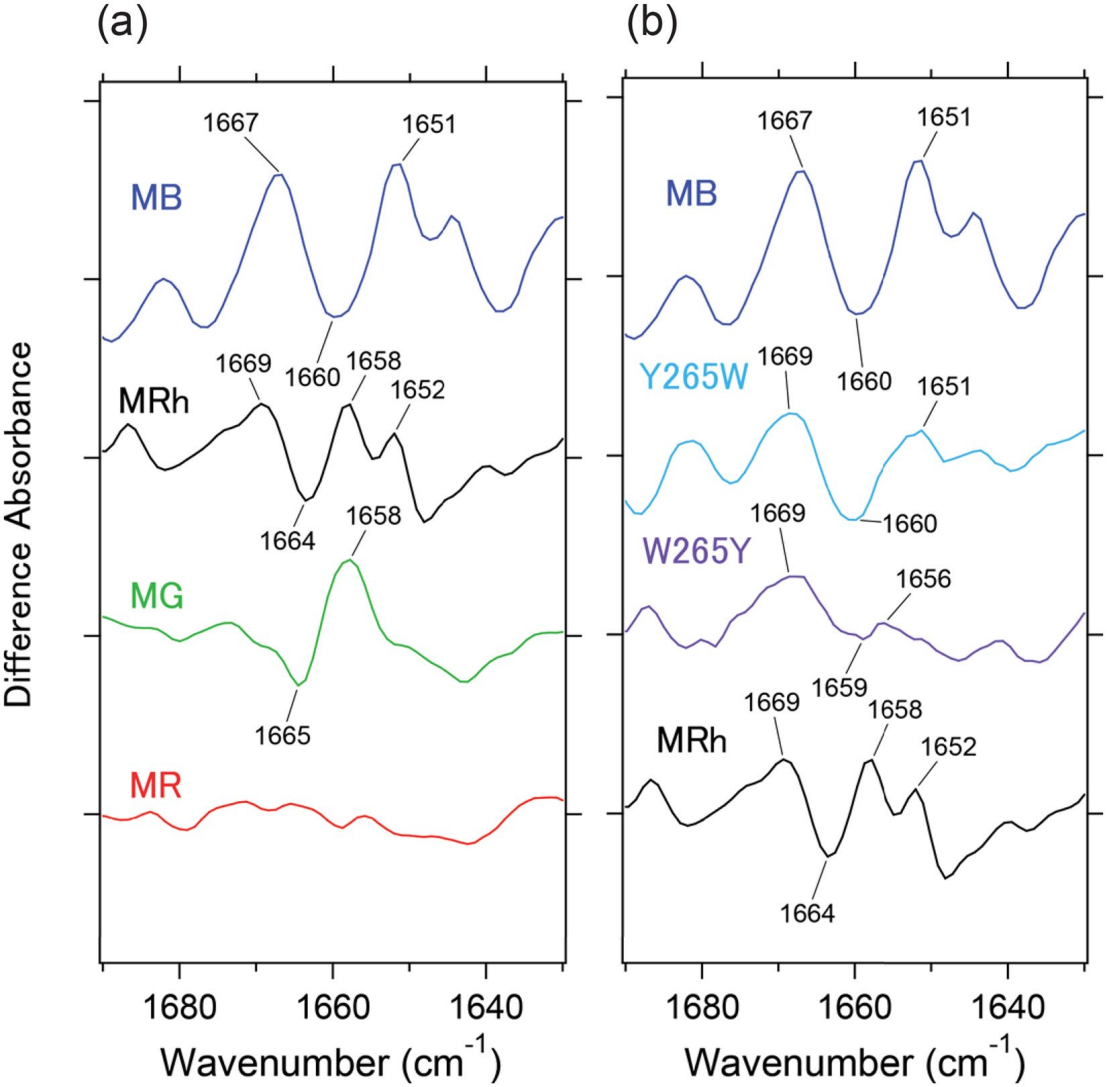
Spectral comparison of the mutant protein in the 1690–1630 cm^−1^ region. (**a**) Expanded spectra from Fig. 1 in the 1690–1630 cm^−1^ region. One division of the y-axis corresponds to 0.00055 absorbance units. (**b**) Light-induced all-*trans* minus 11-*cis* difference FTIR spectra of MB, MB-Y265W, MRh-W265Y and MRh in the 1000–800 cm^−1^ region measured at 77 K in D_2_O. One division of the y-axis corresponds to 0.00055 absorbance units.

Figure 3 shows the different FTIR spectra in the 1700–1620 cm^−1^ range, which mainly monitors the C=O stretch of the peptide backbone (amide-I vibration). Although the band intensity of the amide-I vibration is generally weak, which is in agreement with the comparison of the two protein conformations before and after retinal isomerization, MB showed remarkably strong peaks in the amide-I frequency of the α-helix at 1660 (−)/1651 (+) cm^−1^. This suggests that retinal photoisomerization accompanies large structural changes in α-helices, which correspond to approximately nine amino acid residues that were estimated by a calculation of the band intensity at 1660 cm^−1^ of MB in Fig. 3 and extinction coefficient of the α-helix^29^. Note that the band intensity of MB-Y265W dramatically decreased compared with the MB-WT, which indicates that only a single mutation at position 265 alters the environment of the retinal binding pocket between MB and other pigments. In the crystal structures of both, rhodopsin^22^ and its batho-intermediate^23^, W265 is highly conserved for class-A GPCRs and plays a role as a part of the agonist binding site^30^ that faces in the direction of the C_11_ and C_12_ atoms. Consequently, the structure of the retinal chromophore is curved and allows a fast rate of retinal isomerization into the extended all-*trans* form (Figure S3b). Thus, we postulate that the MB-Y265W mutation reduces the constraint along the polyene chain of retinal, which results in a planar conformation of the chromophore.

Interestingly, the spectral feature of the amide-I band for MRh-W265Y does not reproduce the large structural change in the α-helix compared with MB. Rather, this result seems reasonable because MB and MRh share a sequence identity of only 42% in the retinal binding pocket (Figure S1). Thus, the retinal binding pocket is specially designed to achieve an efficient photoconversion for each pigment. Furthermore, it is known that the corresponding N-H stretch of the peptide backbone (amide-A vibration) appears in the 3400–3200 cm^−1^ region. In Figure S5, MB shows dominant pair bands at 3278 (−)/3248 (+) cm^−1^, whose intensity is affected by the Y265W mutation (light blue line), which is consistent with the observation in the amide-I region.

### Comparison of the X-D stretching vibrations of MB, MRh, MG and MR

**Figure 4:**
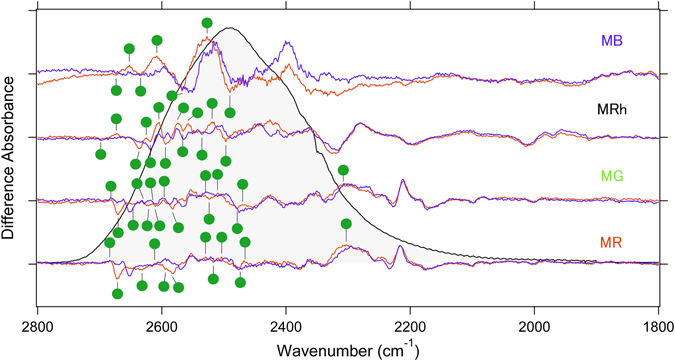
Spectral comparison of the X-D stretching vibrations of MB, MRh, MG and MR in the 28000–1800 cm^−1^ region at 77 K. Red and blue lines represent the spectra in D_2_O and D_2_^18^O, respectively, and green labeled frequencies correspond to those identified as water-stretching vibrations. The gray curve in the 2700–2000 cm^−1^ regions represents O-D stretching vibrations of D_2_O at room temperature. One division of the y-axis corresponds to 0.0002 absorbance units.

**Figure 5:**
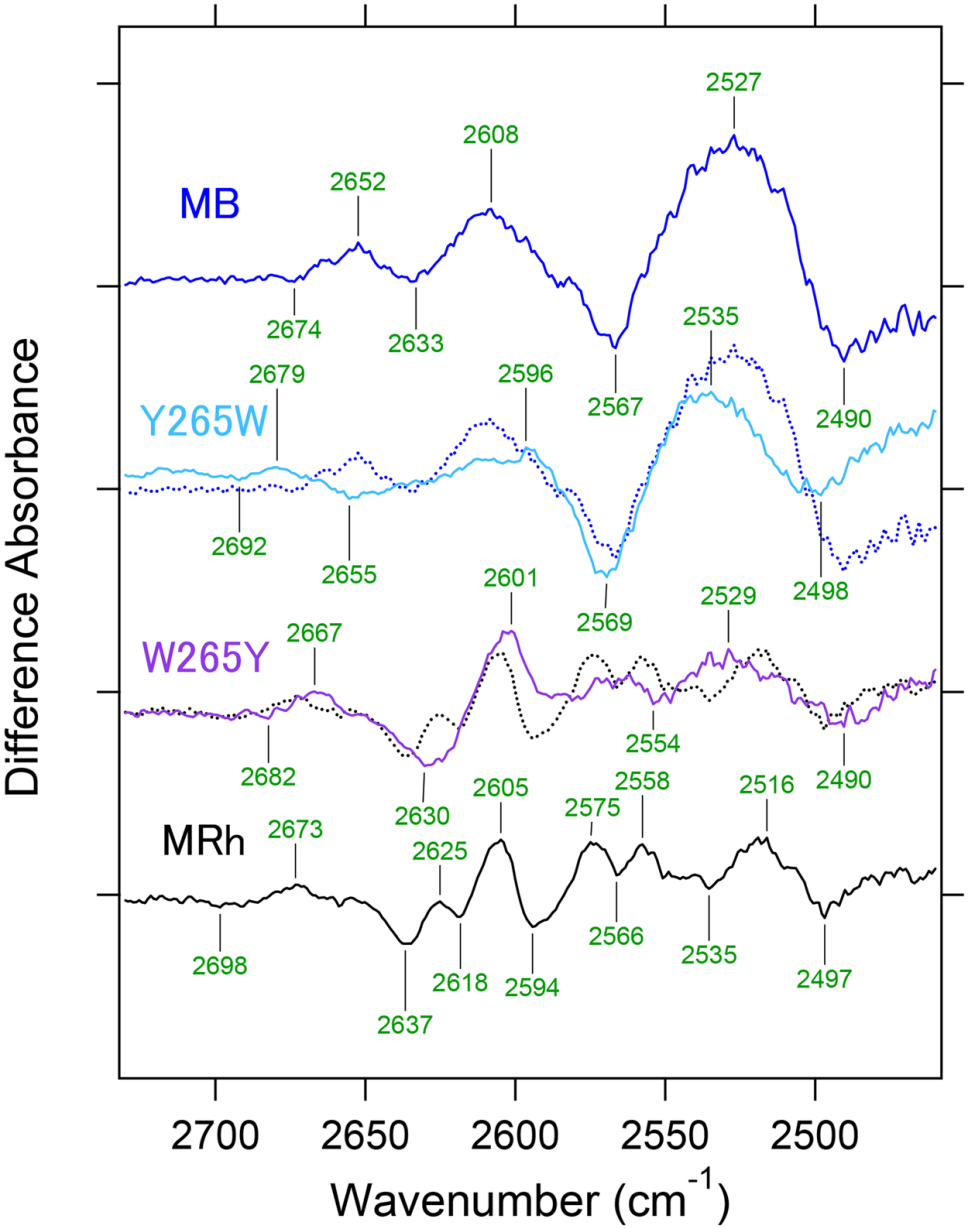
Spectral comparison of the mutant protein in the 2730–2460 cm^−1^ region at 77 K. Light-induced all-*trans* minus 11-*cis* difference FTIR spectra of MB, MB-Y265W, MRh-W265Y and MRh in the 2730–2460 cm^−1^ region measured at 77 K in D_2_O. Blue and black dotted lines correspond to MB and MRh, respectively. One division of the y-axis corresponds to 0.00016 absorbance units.

Figure 4 shows the X-D stretching frequency region in D_2_O that contains information regarding water hydrogen bonding. The observed water O-D stretch bands in both the dark and batho-intermediate states are defined as four and three, respectively. Notably, the MB-specific water band presents a broad and high intensity peak, which appears in the 2600–2500 cm^−1^ region. These frequencies correspond to a moderate hydrogen bonding strength of water molecules and appear similar to that of pure deuterated water in the form of ice (gray curve from 2800–1800 cm^−1^ in Fig. 4). Since deuterated water ice forms a tetrahedral hydrogen bonding network^31, 32^, the observed unique water signals strongly suggest the presence of a water cluster in the retinal binding pocket of MB. In fact, from a previous theoretical study, additional water molecules that form a hydrogen bonding network between Tyr265 and Glu113 in the vicinity of the retinal chromophore were observed^33^. Therefore, the present result strongly supports the presence of additional water near the retina. In Fig. 5, MB shows four negative and three positive peaks of water molecules that are smaller in number than other pigments, which does not necessarily indicate less internal water molecules in MB because peaks in the 2600–2500 cm^−1^ region are stronger than others and consist of multiple water signals, as described above.

Since the retinal and/or retinal binding pocket has a hydrophobic environment in Rh, the content of water molecules near the retinal chromophore is restricted except for two ordered water molecules: Wat2a and Wat2b. Wat2a involves three hydrogen bonds to Ser186, Cys187, and Glu181, and Wat2b is located near Glu113 and stabilizes the protonated Schiff base of the retinal chromophore^34^. Alternatively, a previous analysis of the water signals for MG and MR indicated that the negative dipole moment of the oxygen side of the water molecule, which was possibly localized at the β-ionone ring side of retinal for MR, would associate with a spectral red-shift in absorbance^15^. The identification of MB water signals using site-directed mutational studies provides several insights into the mechanism of the spectral blue-shift. Figure 5 compares water O-D stretches of wild-type MB and MRh with each mutation at the 265 position. In the spectrum of MB-Y265W, the intensities of MB-specific water peaks at 2527 (+)/2490 (−) cm^−1^ are reduced. Presumably, considering the rhodopsin structure, the introduction of the bulky amino acid tryptophan to position 265 of MB prevents the presence of water molecules in the space near the retinal chromophore. Additionally, this result positively suggests that the MB-specific water cluster is located near residue 265. In the amide-I band region (Fig. 3), MB exhibits a higher signal intensity than other pigments, which corresponds to a larger structural change during retinal photoisomerization, presumably because the water cluster may correlate with the α-helical structural change.

In a previous rhodopsin study, it was postulated that the bands at 2618 or 2640 cm^−1^ originated from either Wat2a or Wat2b O-D stretching modes^35^, and both bands of MRh appear at 2618 and 2637 cm^−1^. Similar O-D stretch bands are also confirmed in MB at 2633 cm^−1^. Interestingly, frequencies of this band are down shifted to 2631 cm^−1^ in MB-E113D (Figure S6), which indicates that at least one water molecule is located near the Schiff base similar to rhodopsin. Moreover, the stretching frequency that corresponds to this band is affected by MB-Y265W, although the band shift is opposite to 2655 cm^−1^. This finding strongly suggests the existence of a water hydrogen bonding network between Tyr265 and Glu113 in MB. In fact, the band feature of the MB-specific broadened water O-D stretch is also slightly changed in MB-E113D (Figure S6). Notably, the spectrum of MRh-W265Y provides a similar water signal to that of MB. The O-D stretching vibrational bands at 2637 or 2618 cm^−1^, which originate from Wat2a or Wat2b, respectively, are apparently shifted to 2630 cm^−1^. These results also indicate the presence of a water hydrogen bonding network between Trp265 and Glu113, even though rhodopsin does not have water molecules along the retinal polyene chain. It is conceivable that a reduction in the steric bulkiness from tryptophan to tyrosine at position 265 may alter the environment between MRh and MRh-W265Y.

The spectral comparison of the X-H stretching vibration provides further structural insights into the water containing hydrogen bonding network. Under D_2_O hydration, vibrational bands in the 4000–2700 cm^−1^ region (X-H stretching vibration) originate from H-D, which are unexchangeable O-H and N-H stretching vibrations of amino acids that are involved in a hydrophobic environment. The sharp pair bands at 3485 (+)/3467 (−) cm^−1^ in MRh originate from the O-H stretch of Thr118^14, 36^, and similar bands at 3485 (+)/3428 (−) cm^−1^ in MG and 3486 (+)/3432 (−) cm^−1^ in MR are expected because of the O-H stretches of the corresponding Ser residues (Figure S7). Similarly, in MB, the pair bands at 3521 (+)/3463 (−) cm^−1^ correspond with other pigments in Figure S7. Note that the frequency at 3463 cm^−1^ in the dark state of MB is almost identical with MRh (3467 cm^−1^), which indicates a similar environment around Thr118. Thr118 is conserved in all vertebrate rhodopsins (Rh1 family) and also all short-wavelength sensitive pigments, which belong to the SWS1 family with λ_max_ in the 355–445 nm^37^ range. The crystal structure of rhodopsin shows that Thr118 is one of the residues that form the retinal binding pocket (Figure S3b). Since Thr118 is deeply embedded inside the pocket that is surrounded by hydrophobic residues, it is inaccessible to the internal and/or bulk water, which is in good agreement with the H-D unexchangeable O-H stretching vibration of the Thr118 side chain from FTIR results^38^. Additionally, Glu113 exists in a position 7.8 Å away from Thr118 on the same Helix-III (Figure S3b). Therefore, the observed water clusters in MB may not be located near Thr118 but rather the retinal Schiff base side.

### Molecular mechanism of spectral tuning for primate blue-sensitive visual pigment

**Figure 6:**
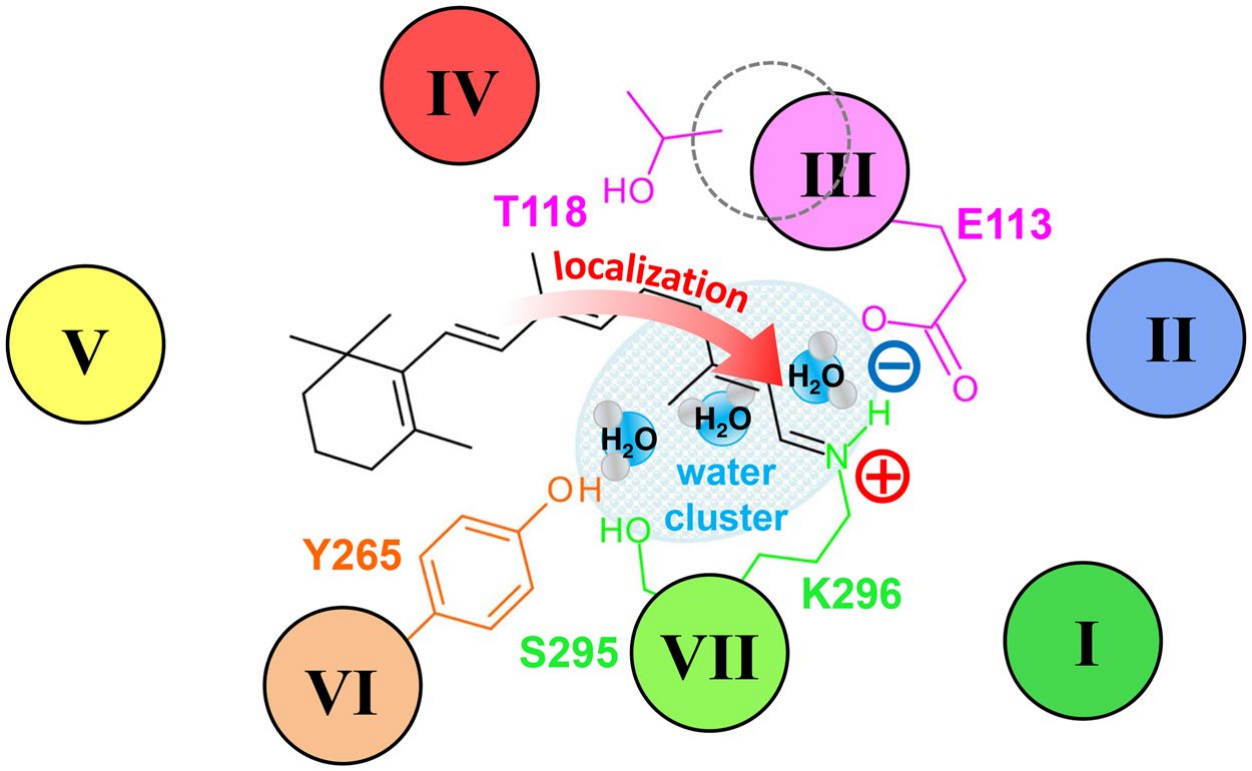
Structural model of the retinal binding pocket in MB that leads to the spectral blue-shift of λ_max_. MB-specific water cluster is situated in the vicinity of retinal Schiff base using a unique hydrogen bonding network with two key residues: Y265 and E113. Consequently, π-electrons along the polyene chain is highly localized towards the protonated Schiff base, which causes the spectral blue-shift of λ_max_ in MB.

Regarding the spectral tuning mechanism of visual pigments, it is necessary to understand the unique interaction of the retinal chromophore with their protein moieties. Vibrational spectroscopies, such as resonance Raman and infrared, have been widely utilized for retinal configuration and retinal-protein interaction investigations. However, less is known about MB due to the difficulties in sample preparation and handling^39^. Previously, Sakmar’s group performed Raman spectroscopy on blue-rho chimera, in which nine amino acids were swapped between the blue pigment and rhodopsin, and determined that its spectral features resembled the spectrum of the protonated Schiff base in a methanol solvent^17^. Thus, they inferred that the three introduced serine residues, G90S, A292S, and A295S, produced a more hydrophilic environment for the retinal Schiff base, which is similar to methanol. Additionally, these amino acids that were placed in the vicinity of the retinal Schiff base caused a reduction in π-electron delocalization along the polyene chain. However, based on the systematic sequence comparison, the serine residues at positions 90 and 295 were completely conserved in primates (including human blue pigment), whereas the residue at position 292 is alanine for all of the primate blue pigments, except for human (serine). Hence, the mechanism underlying the spectral blue-shift of primate blue pigment has been controversial. Based on FTIR studies and mutagenesis analyses, we have provided a novel model for the spectral tuning mechanism of primate blue pigment, as shown in Fig. 6.

In spectral tuning, the polarity around the retinal β-ionone ring and polyene chain is a key factor, and water molecules also play a role in polarity^7^. The observed MB-specific water O-D stretching band in the 2500 cm^−1^ region (Fig. 4) has been assigned to a water cluster that was located in the vicinity of the retinal chromophore. Moreover, this water cluster is localized in between two residues at position 265 and 113 because this water band is affected by both Y265W and E113D mutations. Alternatively, the environment around Thr118 in MB is similar to rhodopsin because of a similar O-H vibrational frequency at 3463 cm^−1^, which originates from Thr118 that is exhibited in the unphotolized state (Figure S7). Combined with our FTIR observations and the case of spectral blue shift by the Ser295 mutation described in the previous study^18^, Fig. 6 shows that both the polarity of the hydrophilic amino acid (like Ser295 and water molecules) synergistically reduced π-electron delocalization along the retinal polyene chain, which successfully led to the spectral blue shift of λ_max_ in MB.

It is known that the vibrational HOOP mode of the retinal chromophore generally influences the chromophore distortion^40, 41^. However, these modes do not influence the wavelength because they monitor local out-of-phase modes that intensify upon retinal geometric distortion^19–21^. Figure S4a,b show the linear correlation between the C_11_=C_12_ HOOP mode frequency and λ_max_ among all pigments, including MB, for both dark and batho-intermediate states. These observations indicate that among π-conjugation systems along the polyene chain of the chromophore, only one double bond at C_11_ and C_12_ influences the electronic state of the retinal chromophore. For MB, we observed the C_11_=C_12_ HOOP mode for the dark state at 961 cm^−1^. This frequency and band shape were considerably distinct for all pigments, as shown in Fig. 2. Intriguingly, in Figure S4c, the relationship between the band area of the C_11_=C_12_ HOOP mode and λ_max_ for each pigment was linear, which is consistent with the results of their frequency-λ_max_ relationships (Figure S4a,b). Because the band shape generally reflects the vibrational coupling, the C_11_=C_12_ HOOP mode in MB appears to be strongly coupled (unlike other pigments), which indicates a geometric planar structure at the C_11_ and C_12_ atoms in the dark state. Although our results provide a plausible explanation that one double bond affects the electronic state that leads to vibrational frequency changes, additional studies with experiments and theoretical calculations^42^ will be required to fully understand the molecular vibrations that cause spectral tuning in cone pigments.

Even if the extent of distortions in unphotolyzed 11-*cis*-retinal for each pigment is variable (Figs 2 and S4), *cis*-*trans* photoisomerization commonly occurs in all pigments (Figure S4d). This can be accomplished by the specific interaction of the retinal chromophore with the protein moiety for all pigments. In fact, peptide-backbone alteration is significantly large in MB after retinal isomerization as compared with other pigments (Fig. 3). Notably, the MB-Y265W mutation led to a significant decrease in amide-I band intensity, such as other pigments. Trp265 is one of three conserved aromatic residues on helix 6 in all class-A GPCRs, and previous solid-state NMR measurements revealed that the interaction between Trp265 and the retinal chromophore stabilizes rhodopsin in the dark state conformation^41^. Moreover, NMR studies implied that Trp265 is a key residue that triggers the opening motion of helix 6 after the photoisomerization of the retinal chromophore^43^. Similar tryptophan-chromophore interactions in a similar position on a helix (F-helix) have also been observed in bacterial rhodopsin for both bacteriorhodpsin (bR) and halorhodopsin (hR) possessing functions as a light-driven proton or chloride pumps, respectively^44^. Moreover, the mutation of Y265W in blue pigment results in a significant spectral red-shift in λ_max_ in the rhodopsin direction^18^. Therefore, we speculate that the observation of a large helical structural change in MB may originate from perturbations in helix 6 during *cis-trans* isomerization.

It is known that hydrogen bonding strength of the Schiff base is important for spectral tuning in relation to the electrostatic interaction between the Schiff base and counterion^7^. In D_2_O, the Schiff base N-D stretch appears in the 2150–1930 cm^−1^ region in Figure S8, where the frequency is decreased as the hydrogen bond strengthens^11^. Recently, our FTIR study successfully identified the Schiff base N-D stretch of MG at 2099 cm^−1^ using a C_15_-D-substituted retinal derivative, which showed a slight down-shift upon retinal isomerization^16^. Importantly, MR had an identical peak pair at the same frequency, and we concluded that the hydrogen bonding strength of the Schiff base of MG and MR was identical. Additionally, the bands at 2013 and 1971 cm^−1^ were tentatively assigned to N-D stretching vibrations of the retinal Schiff base for rhodopsin from the previous report^35^. These frequencies are much lower than those of MG and MR, which is consistent with the stronger hydrogen bond of the Schiff base compared with those in MG and MR, and the spectral blue shift in λ_max_. In the present study, MB shows peaks at 2050–2000 cm^−1^, which are possibly N-D stretching vibrations. If these peaks originate from the Schiff base N-D stretch, then the hydrogen bonding of the Schiff base in MB is stronger than in MG and MR, but slightly weaker than in MRh. This result suggests that electrostatic interactions in the Schiff base region of MB contributes to spectral blue-shift compared with MG and MR, but is similar to MRh. Then, two important structural aspects: the retinal distortion and the polarity of water molecules around the β-ionone ring and polyene chain, contribute to the ~80 nm blue-shift relative to rhodopsin. Therefore, it will be interesting to identify the Schiff base N-D stretch in MB, which promotes a better understanding of the proposed spectral tuning mechanism.

In conclusion, the presented FTIR study identifies the signals of protein-bound water molecules in MB and elucidates the presence of a water cluster in the vicinity of the retinal chromophore. This water cluster is localized between Tyr265 and Glu113 because their corresponding water signals are affected by their point mutations in MB. Presumably, the observed water molecules near the retina play a role in increasing the polarity, which leads to the characteristic spectral blue shift in λ_max_ of MB. Furthermore, in the HOOP mode region at 1000–800 cm^−1^ where signal intensity increases upon retinal distortion, the coupled C_11_=C_12_ HOOP mode frequency exhibits a linear correlation with λ_max_ among all pigments (including MB) for both unphotolyzed and photolyzed states. This finding suggests that *cis*-*trans* photoisomerization commonly occurs in all pigments. However, the retinal geometry in MB possesses the best planar structure as compared with other pigments, as indicated by the spectral sharpness of the C_11_=C_12_ HOOP mode in the unphotolyzed state. Interestingly, MB shows large spectral change in the amide-I vibration of the α-helix upon retinal isomerization, which strongly indicates that retinal isomerization is accompanied by large structural changes in α-helices. Presently, we cannot identify the protonated Schiff base N-D stretch, which typically provides information regarding the hydrogen bonding strength of the Schiff base. Furthermore, spectroscopic analysis by extensive mutations and isotope labeling will enable us to understand the retinal-protein interaction involved in our spectral tuning mechanism.

## Methods

The cDNA of monkey blue (MB) was tagged using the Rho1D4 epitope sequence and introduced into the expression vector pcDNA3.1. This vector was expressed in the HEK293T cell line and regenerated with 11-*cis*-retinal^14, 15^. The regenerated sample was solubilized with a buffer that contained 2% (w/v) n-dodecyl-β-D-maltoside (DDM), 50 mM HEPES, 140 mM NaCl, and 3 mM MgCl_2_ (pH 6.5) and purified by adsorption on an antibody-conjugated column. Then, the purified sample was eluted with a buffer that contained 0.12 mg/mL 1D4 peptide, 0.02% DDM, 0.004% cholesteryl hemisuccinate (CHS), 50 mM HEPES, 140 mM NaCl, and 3 mM MgCl_2_ (pH 6.5).

Site-directed mutagenesis was performed using the Quik Change Multisite-Directed Mutagenesis Kit (Agilent Technologies, Inc., Santa Clara, CA, USA)

For the FTIR spectroscopy measurements, MB was reconstituted into phosphatidylcholine (PC) liposomes with a protein-to-lipid molar ratio of 1:30 by dialysis to remove DDM. The reconstituted sample was suspended in a buffer that contained 2 mM phosphate and 10 mM NaCl (pH 7.25), placed onto a BaF_2_ window and dried with an aspirator. Low-temperature FTIR spectroscopy was applied to the films that were hydrated with H_2_O, D_2_O, or D_2_^18^O at 77 K, as previously described^10, 45^. For the formation of the batho-intermediate state of MB, the samples were irradiated with 400 nm light (using an interference filter) for 5 min. For the reversion of the batho-intermediate state to the dark state, the samples were irradiated with >520 nm light for 5 min. For each measurement, 128 interferograms were collected and 40 recordings were averaged. FTIR spectra were recorded at a 2 cm^−1^ resolution.

## Electronic supplementary material

Supplementary Information for publication
