# Supplementary material for: Spectral Tuning Mechanism of Primate Blue-sensitive Visual Pigment Elucidated by FTIR Spectroscopy
Source: Sci Rep. 2017 Jul 7;7:4904. doi: 10.1038/s41598-017-05177-4 (PMC5501831; doi:10.1038/s41598-017-05177-4)
Supplement: Supplementary file 1 — Supplementary Information for publication [file 41598_2017_5177_MOESM1_ESM.pdf]

SUPPLEMENTARY INFORMATION

Spectral Tuning Mechanism of Primate Blue-Sensitive Visual Pigment Elucidated by FTIR Spectroscopy

Kota Katayama[1,2,a], Yuki Nonaka[1,a], Kei Tsutsui[3], Hiroo Imai[3], and Hideki Kandori[*,1,2]

[1]Department of Life Science and Applied Chemistry, Nagoya Institute of Technology, Showa-ku, Nagoya 466-8555, Japan

[2]OptoBioTechnology Research Center, Nagoya Institute of Technology, Showa-ku, Nagoya 466-855, Japan

[3]Primate Research Institute, Kyoto University, Inuyama 484-8506, Japan

[*]Corresponding authors: Department of Life Science and Applied Chemistry, Nagoya Institute of Technology, Showa-ku, Nagoya 466-8555, Japan. Phone and fax: 81-52-735-5207. E-mail: kandori@nitech.ac.jp.

[a]These authors contributed equally to this work.

Table 1

Table1: Frequency of HOOP bands (Table.1)					
	(-) C_{11}=C_{12} HOOP	(+) C_{11} HOOP	(+) C_{12} HOOP	Δ (C_{11}-C_{12} HOOP)	S^a
MB	961	928	865	63	0.00252
MB Y265W	963	924	865	59	0.00190
MRh W265Y	970	923	857	66	0.00299
MRh	966	917	857	60	0.00238
MG	973	907[b]	848	59	0.00540
MR	973	906[c]	847	59	0.00516

[a] Sum of the intensity of negative bands from C_{11}=C_{12} HOOP

[b] Average of two positive bands (911cm^{-1} and 903cm^{-1})

[c] Average of two positive bands (909cm^{-1} and 903cm^{-1})

Supplementary Figure legends

Figure S1. **Amino acid sequences of monkey blue, rhodopsin (bovine and monkey), monkey green, and monkey red.** (a) 26 amino acids within 5 Å from the retinal chromophore in the X-ray structure of bovine rhodopsin are shown. The amino acids are identical between bovine and monkey rhodopsins. The two key residues for monkey blue, Glu113 and Tyr265 are highlighted by red. The residue numbers are based on the bovine rhodopsin sequence. (b) Schematic representation of the 26 amino acid differences around 11-*cis*-retinal among each pigments.

Figure S2**. Light-induced difference FTIR and UV-visible absorption spectra of mutant proteins, and correlation between the retinal C=C stretching frequency and λ_{max}.** (a) Light-induced difference FTIR spectra of MB, MB-Y265W, MRh-W265Y, MRh, MG and MR in the 1800-800 cm^{-1} region at 77 K. One division of the y-axis corresponds to 0.0013 absorbance units. (b) Expanded spectra from (a) in the 1630- 1450 cm^{-1} region. One division of the y-axis corresponds to 0.0013 absorbance units. (c) UV-visible absorption spectra of MB (λ_{max} = 417 nm), MB-Y265W (λ_{max} = 434 nm), MRh-W265Y (λ_{max} = 481 nm), MRh (λ_{max} = 499 nm), MG (λ_{max} = 530 nm) and MR (λ_{max} = 563 nm). (d) Correlation of the unphotolyzed retinal C=C stretching frequencies of MB (1576 cm^{-1}), MB-Y265W (1566 cm^{-1}), MRh-W265Y (1564 cm^{-1}), MRh (1561 cm^{-1}), MG (1534 cm^{-1}) and MR (1526 cm^{-1}) from (b) with their absorption maxima from (c).

Figure S3. **Structural changes from rhodopsin (green) to bathorhodopsin (red), and representation around retinal binding pocket.** (a) Structural changes from rhodopsin (green) to batho-intermediate state, bathorhodopsin (red) are viewed from extracellular side. The double bond at the position C_{11} and C_{12} (C_{11}=C_{12}) is highlighted about highly distortion. (b) Part of the retinal binding pocket are viewed from helix VI, where the curvature along the polyene chain of retinal is emphasised, and side chain of W265 is facing towards atoms of C_{11} and C_{12}.

Figure S4. **HOOP band correlation diagrams for dark and batho-intermediate state among each pigments with their absorption maxima, and summary of the HOOP band frequencies of the batho-intermediate state.** (a) Correlation of the C_{11}=C_{12} HOOP band frequencies of different pigments in the dark state with their absorption maxima. (b) Correlation of the C_{11} or C_{12} HOOP band frequencies of different pigments in the batho-intermediate state with their absorption maxima. (c) Calculated area of C_{11}=C_{12} HOOP band of different pigments in the dark state with their absorption maxima. (d) Summary of the HOOP band frequencies of different pigments in the batho-intermediate state.

Figure S5. **Light-induced difference FTIR of MB, MB-Y265W, MRh-W265Y and MRh in the 3400-3200 cm^{-1} region at 77 K.** One division of the y-axis corresponds to 0.0005 absorbance units.

Figure S6. **Light-induced difference FTIR of MB (blue solid and dotted lines), MB-Y265W (light blue) and MB-E113D (magenta) in the 2730-2460 cm^{-1} region at 77 K.** One division of the y-axis corresponds to 0.00016 absorbance units.

Figure S7. **Light-induced difference FTIR of MB, MB-Y265W, MRh-W265Y, MRh, MG and MR in the 3600-3380 cm^{-1} region at 77 K.** One division of the y-axis corresponds to 0.0005 absorbance units.

Figure S8. **Light-induced difference FTIR of MB, MRh, MG and MR in the 2150-1930 cm^{-1} region at 77 K.** One division of the y-axis corresponds to 0.00004 absorbance units.

Figure S1

(a)

	Helix III (113–125)	Helix IV (164)	E-II loop (181–191)
monkey blue (417nm) :	E G . . G T . . G L . . G	G	E . . . . S C G P . W
rhodopsin (499nm) :	E G . . A T . . G E . . L	A	E . . . . S C G I . Y
monkey green (530nm) :	E G . . V S . . G I . . L	A	H . . . . S C G P . V
monkey red (563nm) :	E G . . V S . . G I . . L	S	H . . . . S C G P . V

	Helix V (207–212)	Helix VI (261–269; 265)	Helix VII (292–296)
monkey blue (417nm) :	L F . . C F	F . . . Y . . Y A	A F . S K
rhodopsin (499nm) :	M F . . H F	F . . . W . . Y A	A F . A K
monkey green (530nm) :	L M . . C C	F . . . W . . Y A	A Y . A K
monkey red (563nm) :	L M . . C C	Y . . . W . . Y T	A Y . A K

(b)

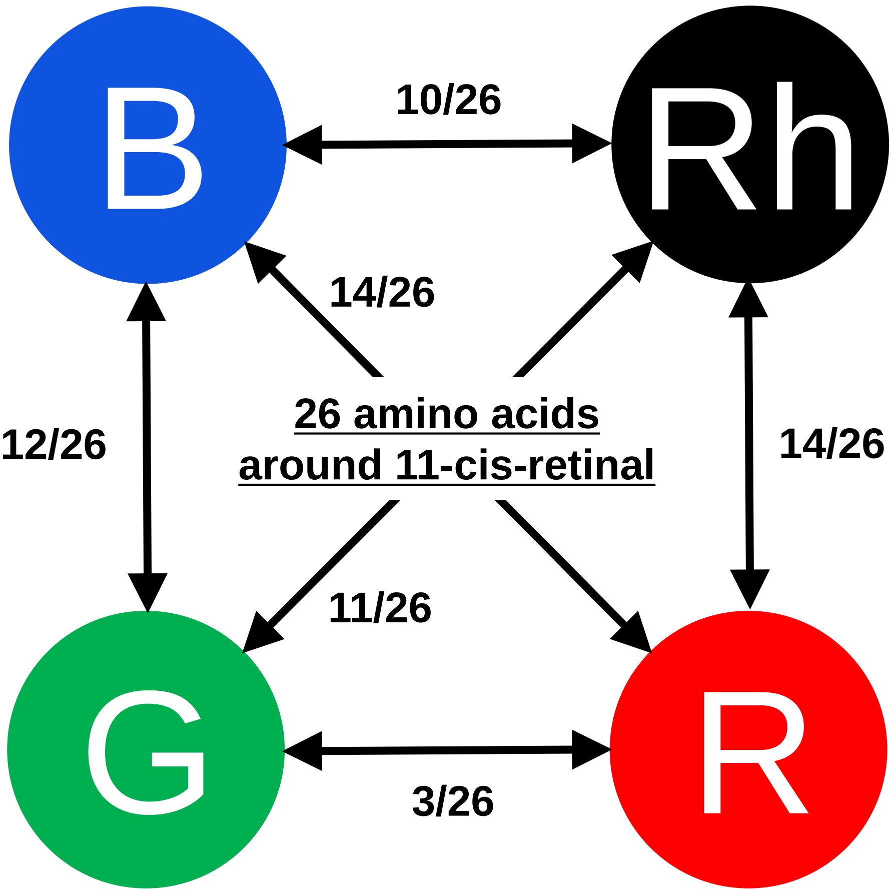

Figure S2

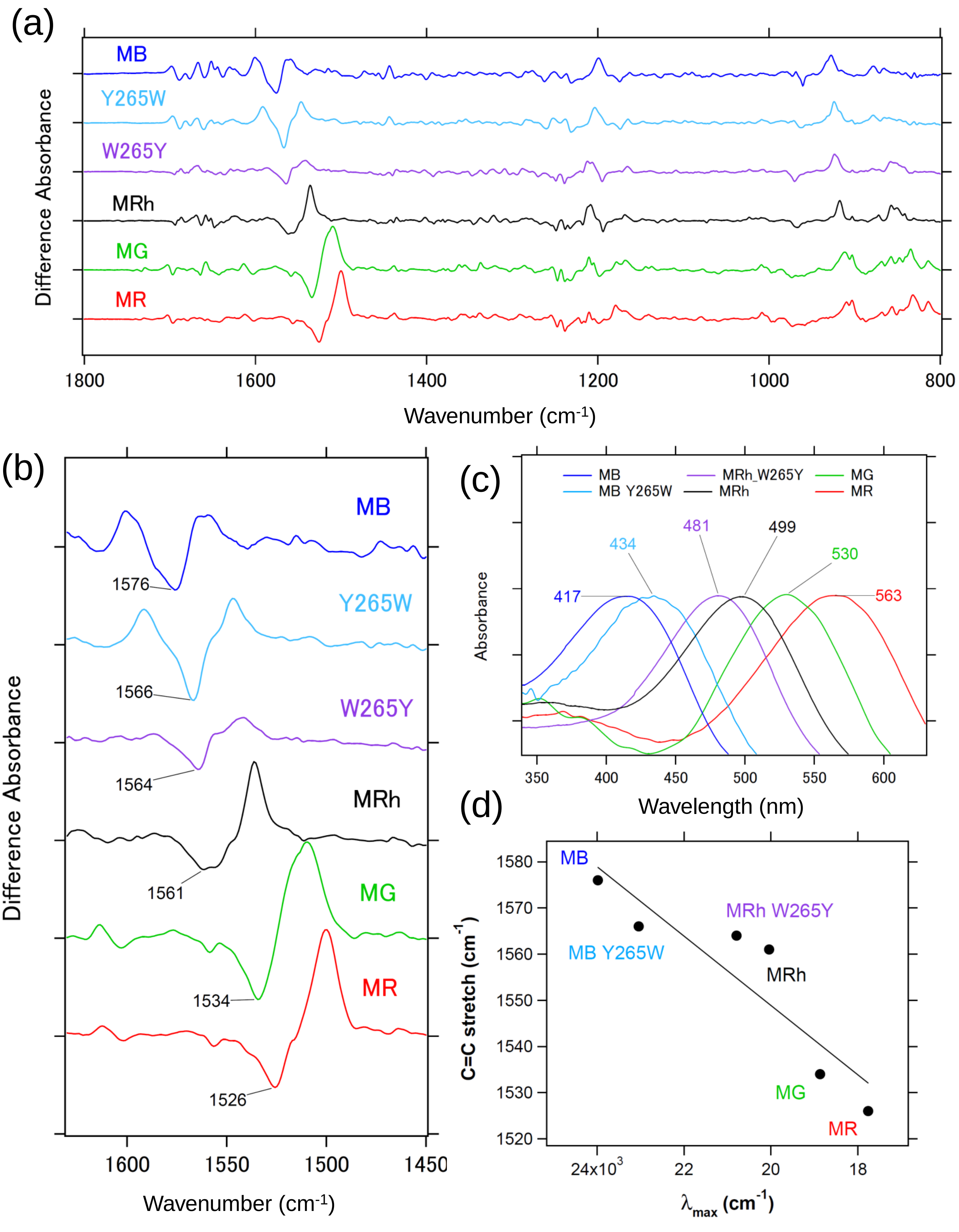

(a)
MB
Y265W
W265Y
MRh
MG
MR
Difference Absorbance
1800
1600
1400
1200
1000
800
Wavenumber (cm-1)
(b)
MB
Y265W
W265Y
MRh
MG
MR
1576
1566
1564
1561
1534
1526
Difference Absorbance
1600
1550
1500
1450
Wavenumber (cm-1)
(c)
MB
MB Y265W
MRh_W265Y
MRh
MG
MR
417
434
481
499
530
563
Absorbance
350
400
450
500
550
600
Wavelength (nm)
(d)
MB
MB Y265W
MRh W265Y
MRh
MG
MR
C=C stretch (cm-1)
1580
1570
1560
1550
1540
1530
1520
24x10^3
22
20
18
λmax (cm-1)

Figure S3

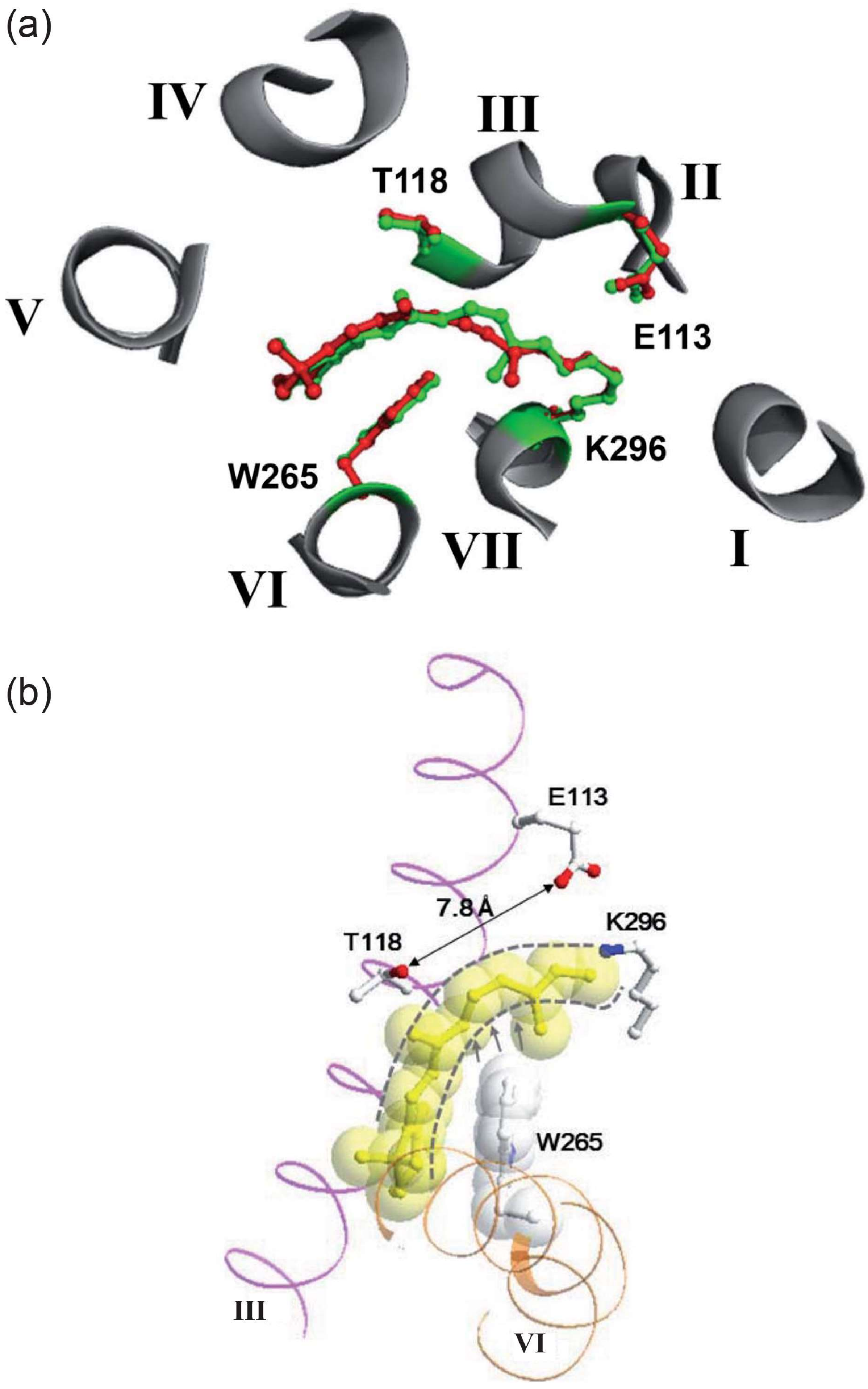
(a)
IV
III
T118
II
V
E113
K296
W265
VII
I
VI
(b)
E113
7.8 Å
K296
T118
W265
III
VI

Figure S4

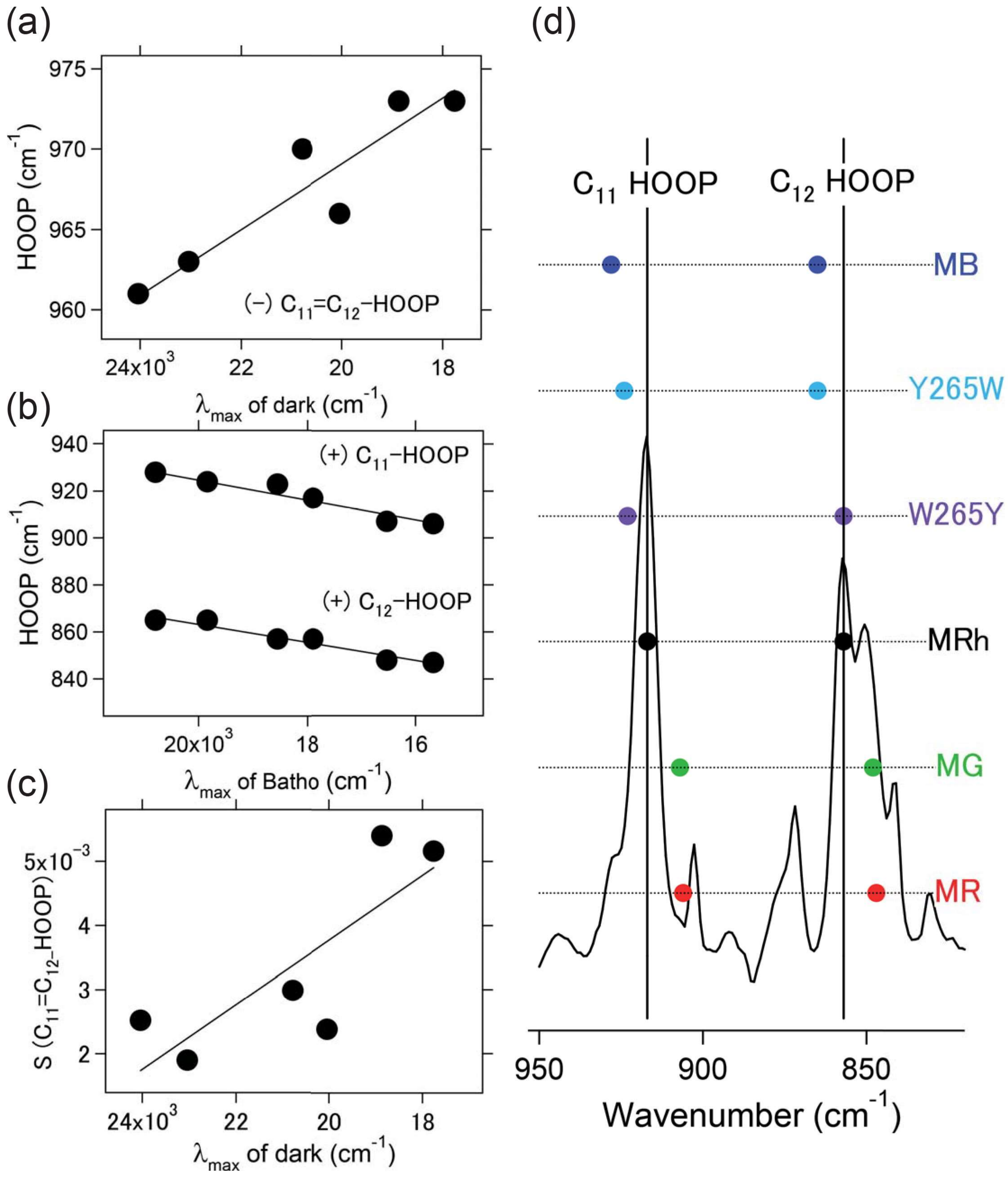

(a)
975
970
965
960
HOOP (cm-1)
(−) C11=C12−HOOP
24x10^3
22
20
18
λmax of dark (cm-1)
(b)
940
920
900
880
860
840
HOOP (cm-1)
(+) C11−HOOP
(+) C12−HOOP
20x10^3
18
16
λmax of Batho (cm-1)
(c)
5x10^-3
4
3
2
S (C11=C12−HOOP)
24x10^3
22
20
18
λmax of dark (cm-1)
(d)
C11 HOOP
C12 HOOP
MB
Y265W
W265Y
MRh
MG
MR
950
900
850
Wavenumber (cm-1)

Figure S5

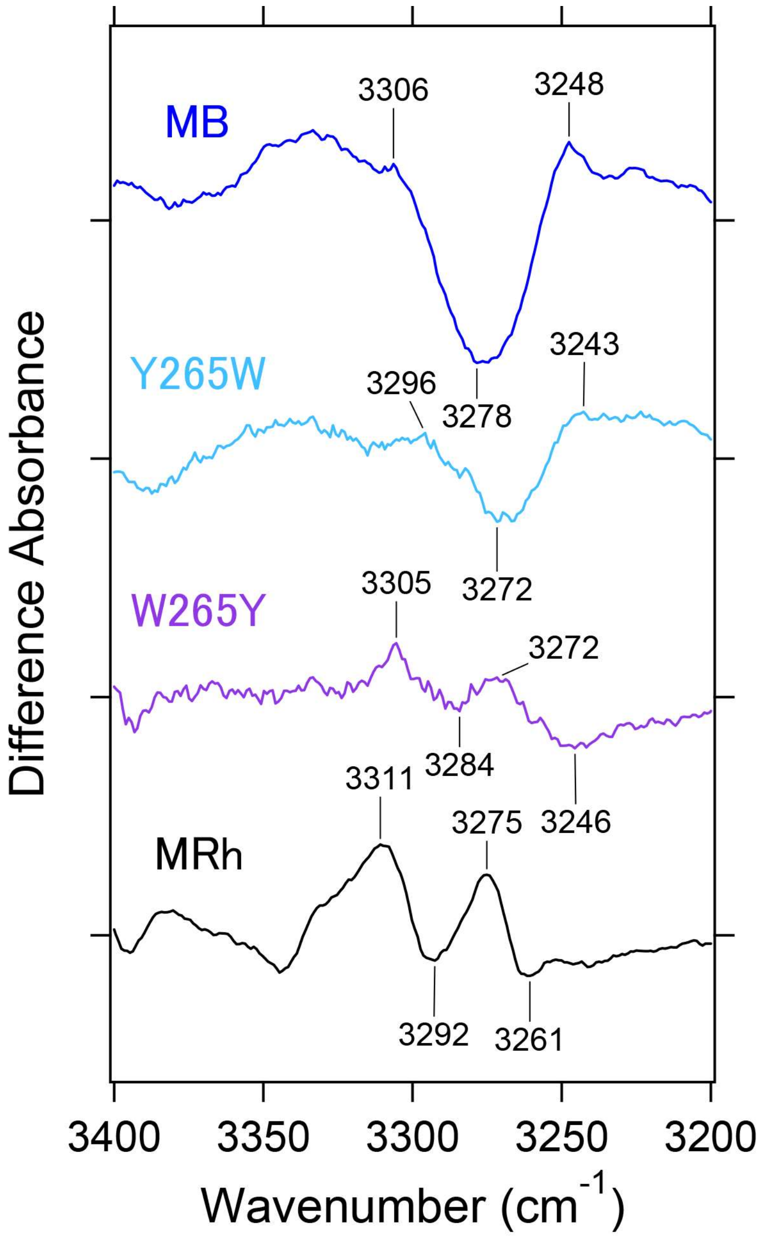
MB
3306
3248
3278
Y265W
3296
3243
3272
W265Y
3305
3272
3284
3246
MRh
3311
3275
3292
3261
Difference Absorbance
3400
3350
3300
3250
3200
Wavenumber (cm-1)

Figure S6

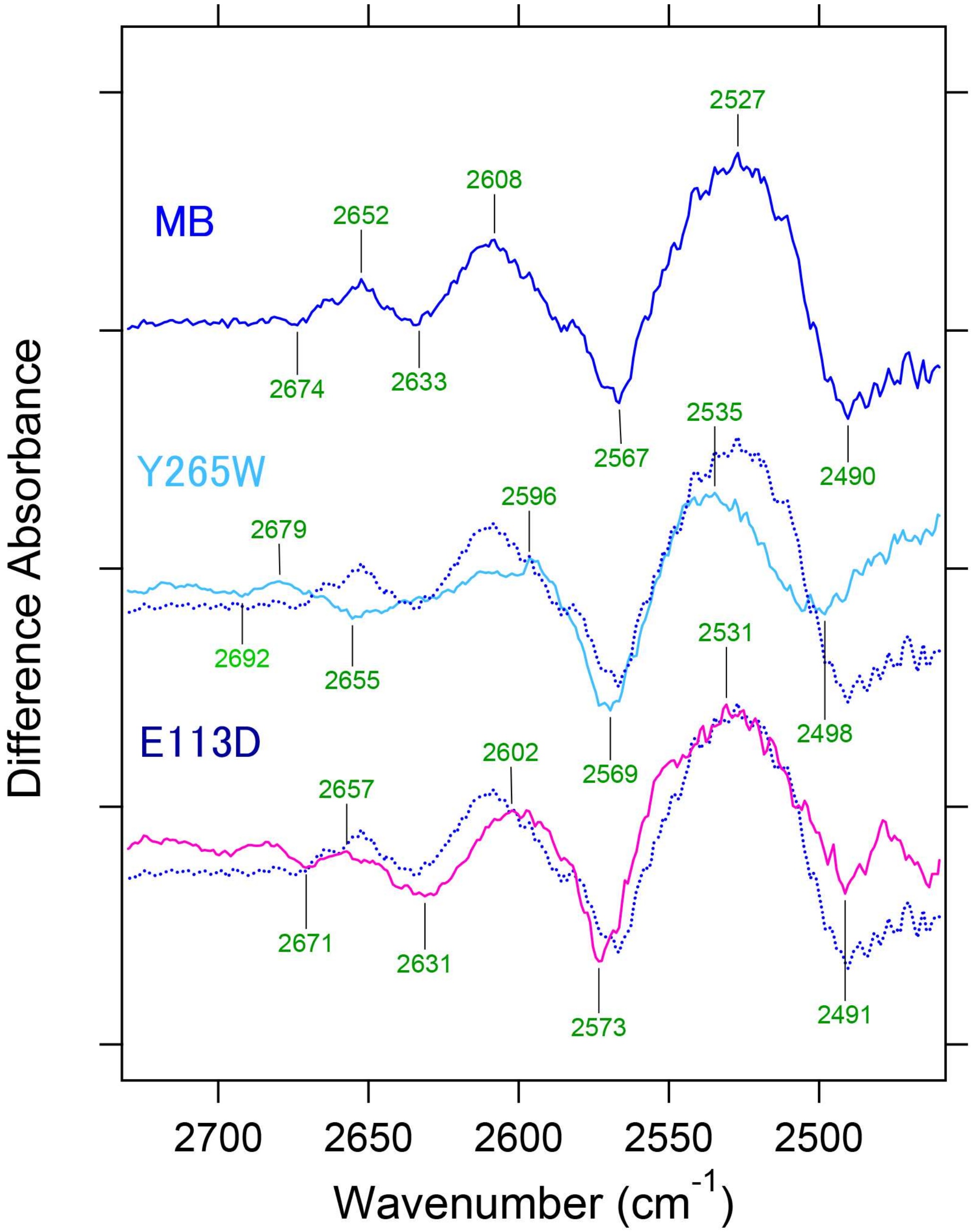

MB
2527
2608
2652
2674
2633
2567
2490
Y265W
2535
2596
2679
2692
2655
2569
2498
E113D
2531
2602
2657
2671
2631
2573
2491
2700
2650
2600
2550
2500
Wavenumber (cm-1)
Difference Absorbance

Figure S7

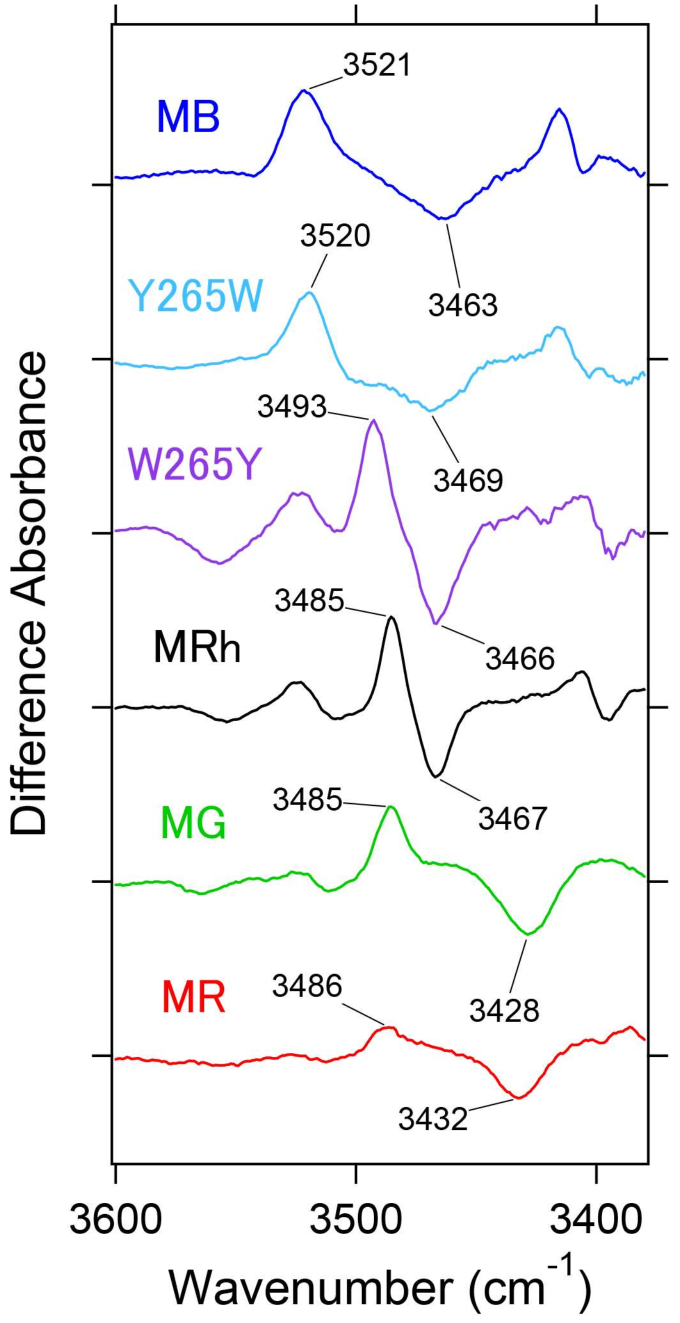
MB
3521
3463
Y265W
3520
3469
W265Y
3493
3466
MRh
3485
3467
MG
3485
3428
MR
3486
3432
Difference Absorbance
3600
3500
3400
Wavenumber (cm-1)

Figure S8

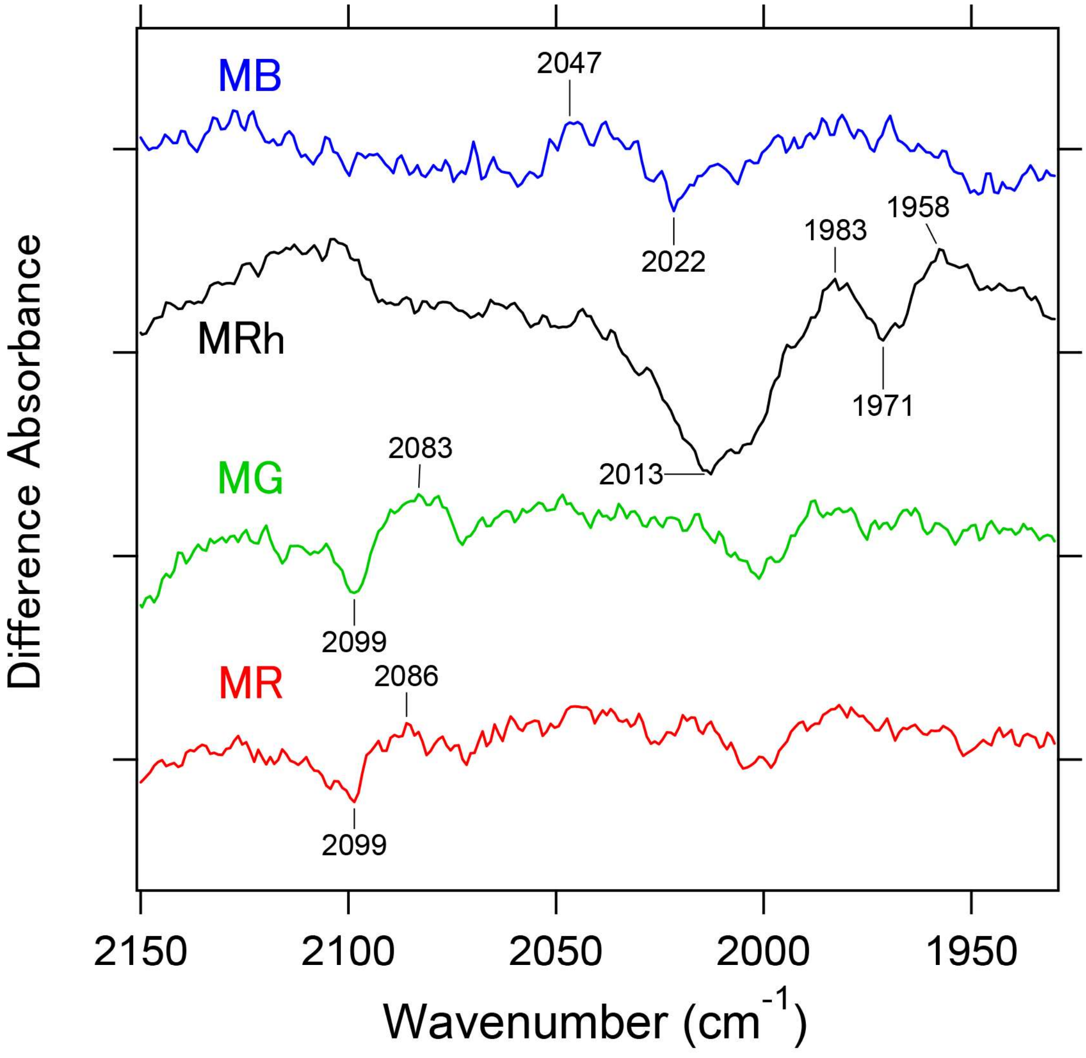
MB
2047
2022
MRh
1983
1958
1971
2013
MG
2083
2099
MR
2086
2099
Difference Absorbance
2150
2100
2050
2000
1950
Wavenumber (cm-1)
